# The effects of simvastatin‐loaded nanoliposomes on human multilineage liver fibrosis microtissue

**DOI:** 10.1111/jcmm.18529

**Published:** 2024-07-10

**Authors:** Shima Parsa, Maryam Dousti, Nasim Mohammadi, Mozhgan Abedanzadeh, Niloofar Dehdari Ebrahimi, Mahintaj Dara, Mahsa Sani, Muhammad Nekouee, Samira Sadat Abolmaali, Farnaz Sani, Negar Azarpira

**Affiliations:** ^1^ Shiraz Institute for Stem Cell & Regenerative Medicine Shiraz University of Medical Sciences Shiraz Iran; ^2^ Department of Pharmaceutical Nanotechnology, School of Pharmacy Shiraz University of Medical Sciences Shiraz Iran; ^3^ Transplant Research Center Shiraz University of Medical Sciences Shiraz Iran; ^4^ Stem Cells Technology Research Center Shiraz University of Medical Sciences Shiraz Iran

**Keywords:** KLF2, liver fibrosis, liver microtissue, NAFLD, Nanoliposome, NO, simvastatin

## Abstract

In this in vitro study, for the first time, we evaluate the effects of simvastatin‐loaded liposome nanoparticles (SIM‐LipoNPs) treatment on fibrosis‐induced liver microtissues, as simvastatin (SIM) has shown potential benefits in the non‐alcoholic fatty liver disease process. We developed multicellular liver microtissues composed of hepatic stellate cells, hepatoblastoma cells and human umbilical vein endothelial cells. The microtissues were supplemented with a combination of palmitic acid and oleic acid to develop fibrosis models. Subsequently, various groups of microtissues were exposed to SIM and SIM‐LipoNPs at doses of 5 and 10 mg/mL. The effectiveness of the treatments was evaluated by analysing cell viability, production of reactive oxygen species (ROS) and nitric oxide (NO), the expression of Kruppel‐like factor (KLF) 2, and pro‐inflammatory cytokines (interleukin(IL)‐1 α, IL‐1 β, IL‐6 and tumour necrosis factor‐α), and the expression of collagen I. Our results indicated that SIM‐LipoNPs application showed promising results. SIM‐LipoNPs effectively amplified the SIM‐klf2‐NO pathway at a lower dosage compatible with a high dosage of free SIM, which also led to reduced oxidative stress by decreasing ROS levels. SIM‐LipoNPs administration also resulted in a significant reduction in pro‐inflammatory cytokines and Collagen I mRNA levels, as a marker of fibrosis. In conclusion, our study highlights the considerable therapeutic potential of using SIM‐LipoNPs to prevent liver fibrosis progress, underscoring the remarkable properties of SIM‐LipoNPs in activating the KLF2‐NO pathway and anti‐oxidative and anti‐inflammatory response.

## 1 INTRODUCTION

Non‐alcoholic fatty liver disease (NAFLD), with a prevalence of 32.4% worldwide, is the most common chronic liver disorder.^1^ NAFLD is defined by an accumulation of more than 5% hepatic steatosis in liver tissue, leading to hepatic lesions, inflammation and fibrosis.^2^ Excessive fat accumulation triggers immune cell infiltration and overgeneration of reactive oxygen species (ROS) production, which induces an inflammatory and apoptotic environment in the liver, ultimately leading to non‐alcoholic steatohepatitis (NASH).^3^, ^4^ Moreover, hepatocyte apoptosis exacerbates the inflammatory cascade, while apoptotic bodies further fuel the activation and transformation of hepatic stellate cells (HSCs) into fibrogenic myofibroblasts, ultimately fostering the progression of liver fibrosis. Importantly, activated HSCs play a dual role: not only are they involved in fibrogenesis, but they also participate in the liver's inflammatory response. This contribution occurs through the secretion of pro‐inflammatory cytokines, such as interleukin (IL)‐1, IL‐6 and tumour necrosis factor (TNF)‐α.^5^, ^6^

Investigating gene expression regulation in NAFLD reveals a striking consistency in the activation program of HSCs across multiple etiologies, suggesting a convergent role for HSC activation in the pathogenesis of NAFLD.^7^ The activated HSCs and their transformation into myofibroblasts contribute to extreme collagen type I synthesis, resulting in qualitative changes to the collagenous extracellular matrix (ECM) components, consequently leading to the development of liver fibrosis. The gradual progression of fibrosis can ultimately lead to advanced liver disease, necessitating the need for liver transplantation.^8^ Despite the high prevalence of NAFLD and its life‐threatening consequences, no approved pharmaceutical interventions are presently available for its treatment.^9^, ^10^ As a result, the only proven treatment for a variety of liver diseases is liver transplant, justifying the necessity of novel therapeutic options or drug repositioning to reduce liver damage brought on by NAFLD.^11^

Simvastatin (SIM) is typically administered due to its ability to lower blood cholesterol levels and the expression of LDL receptors in hepatocytes.^12^ SIM has also been reported to exhibit hepatoprotective effects in patients with NAFLD. It has been shown to reduce elevated liver enzymes, mitigate hepatic fatty infiltration and even stabilize or reverse fibrosis by inhibiting HSC proliferation.^13^, ^14^, ^15^, ^16^ A recent study has also demonstrated SIM's capacity to increase antioxidant enzyme activity and diminish lipid peroxidation, which can improve liver function in NAFLD.^17^, ^18^ SIM is shown to be the most efficient statin safeguarding the hepatic endothelium. It is suggested this agent plays its role through activation of the transcription factor Kruppel‐like factor (KLF2)‐nitric oxide (NO) pathway leading to hepatic endothelial protection.^19^, ^20^

Nevertheless, the utilization of SIM is constrained by disputes surrounding its impact on liver functionality. Despite SIM's demonstrated protective properties on NAFLD by Wang et al., adverse effects on the liver remain a notable concern associated with statin therapy.^16^, ^21^ In this regard, SIM‐loaded liposome nanoparticle (SIM‐LipoNP) is a potential choice for therapeutic use due to its stability and sustained release profile.^22^ Liposomal delivery can decrease the required dosage of the intact SIM.^23^ Modifying the active drug's distribution through a liposomal drug delivery system represents a promising approach to mitigate side effects and enhance treatment efficacy.^24^ However, studies on the impact of SIM‐encapsulated nanoliposomes on NAFLD have yet to be achieved.

In order to pathologically investigate NAFLD, animal models, despite their usefulness, are limited in capturing all aspects of human NAFLD.^25^, ^26^ The recent food and drug administration announcement exempting certain novel therapies from animal testing marks a significant development in drug development practices. This shift has the potential to pave the way for further exploration of alternative testing methods.^27^ Therefore, in vitro models of human fibrosis in two‐dimensional (2D) and more complex 3D cultures have been introduced.^28^, ^29^ Recognizing the diverse cellular contributions to NAFLD pathogenesis, the inclusion of representative parenchymal (HepG2s) and non‐parenchymal, endothelial cells (HUVECs) and hepatic stellate cells (LX‐2 s) is crucial for in vitro NAFLD models that accurately reflect the human liver microenvironment. Consequently, a microtissue comprising these cell types presents an optimal platform for investigating pharmacological interventions, such as SIM‐LipoNP, on fibrosis process, particularly within the context of HSC and endothelial cell activity.

## 2 METHODS

### 2.1 Preparation and characterization of SIM‐LipoNPs

SIM‐LipoNPs were formulated using the freeze‐drying method as described elsewhere.^30^ Briefly, 1,2‐distearoyl‐sn‐glycero‐3‐phosphocholine (DSPC) and cholesterol at a 2:1 molar ratio were dissolved in deionized H2O: tert‐Butyl alcohol (1:1), following the addition of SIM at 1 to 10 molar ratio of drug to total lipid. Empty liposome was also formulated with the same lipid molar ratios. The final solution was frozen at −80°C and dried under vacuum to obtain a uniform powder. The lyophilized products were stored at −20°C. The final lyophilized powder was reconstituted by adding sterile phosphate‐buffered saline (PBS), (at 60°C) to reach the desired concentration and stirred mildly at 60°C for 4 h to form an aqueous liposome suspension. A bench‐top extruder was used to obtain a homogenous liposome suspension with uniform particle size and a narrow polydispersity index. So, the aqueous suspension of liposome was extruded through polycarbonate filters with a pore diameter of 400 nm, at 60°C, for five cycles. The average liposome particle size and the size distribution, with or without SIM, were measured using a Nanoparticle Size Analyzer (NANOTRAC WAVE, Microtrac, Germany), which was carried out at room temperature. In addition, the zeta potential of the liposome with or without SIM was measured using ZETA‐check (Microtrac, Germany). To confirm the particle size uniformity of liposomes and also their spherical shape, Atomic Force Microscopy (AFM) was utilized using the non‐contact mode (JPK BioMAT Workstation).

### 2.2 Investigation of cellular uptake of liposomes

Labelled liposomes were prepared to follow the cellular uptake of liposomes. 1,2‐Dipalmitoyl‐sn‐glycero‐3‐phosphoethanolamine (DPPE) was conjugated to the Fluorescein isothiocyanate (FITC). At first, DPPE and FITC with 1:1.5 molar ratios were dissolved in chloroform following the addition of Triethylamine (TEA) with a 3‐fold molar ratio of DPPE. The solution was stirred at 50°C overnight. The reaction solution was dried under vacuum conditions, and the resultant was dispersed in DMSO and dialyzed against deionized water to remove the unreacted FITC. Finally, the final labelled phospholipid was freeze‐dried and added to the freeze‐dried lipid materials to reach 1% of the lipid part of the final liposomes.

HUVECs, LX‐2s and HepG2s were cultured in 3D culture types at a ratio of 1:1:3, respectively, and used to study labelled liposomes' cellular uptake. Cells were seeded in 24‐well plates at a density of 5000 cells per well in complete medium (Roswell Park Memorial Institute (RPMI) 1640, 10% fetal bovine serum (FBS), 1% penicillin and streptomycin) and incubated at 37°C, 5% CO_2_ for 24 h. On day 4 of cell culture, the medium was replaced with a fresh growth medium containing 25 μg/mL FITC labelled liposomes, and cells were analysed after incubation for 24 h using fluorescent microscopy.

### 2.3 In‐vitro drug release

SIM release from liposomal formulation was studied using the dialysis method through cellulose membrane in 10 mM phosphate buffer (pH 7.4). Briefly, 1 mL of SIM‐LipoNPs was poured into a dialysis bag (with 2 kDa cut‐off) following the insertion of the bag into 10 mL of release media. SIM release was studied at 37°C while stirring at 350 rpm. The release medium was removed at different time intervals, and the fresh medium was replaced. The samples were freeze‐dried, and the amount of released drug was determined after dissolution in a mixture of methanol and water (1:1) using the ultraviolet (UV) spectroscopy method.^22^, ^31^ To explore how the inclusion of SIM in liposomes affects its release profile, an equal amount of free SIM at 0.5% Sodium dodecyl sulfate (SDS) was poured into a dialysis bag (with a 2 kDa cut‐off), and drug release kinetics were investigated using a comparable approach.

### 2.4 Cell culture conditions

The hepatoblastoma cell line (HEPG2), hepatic stellate cell line (LX‐2) and endothelial cell line (HUVEC) were provided by Pasture Institute (Iran, Tehran). HEPG2s and LX‐2s were routinely cultured in Roswell Park Memorial Institute (RPMI) 1640, 15 mM HEPES, [+] Glutamax R2622 (Shellmax). HUVECs were cultured in Dulbecco's Modified Eagle Medium/Nutrient Mixture F‐12 (DMEM/F12, Gibco, USA). Both of the mediums were supplemented with Penicillin–Streptomycin (100 U/mL) (Bioidea, Iran) and 10% (vol/vol) FBS (Gibco, USA). All cells were cultured in each medium at 37°C in 5% CO_2_ (exchanging the culture media every other day). On their third passage, the cells were trypsinized (0.05% trypsin/Ethylenediamine tetraacetic acid (EDTA), Shellmax, China) and ready for further experiments.

### 2.5 Multicellular liver microtissue (spheroids) development

5000 cells co‐culture of the three mentioned cell lines was used to develop microtissues at the ratio of 3:1:1 for HepG2s+LX‐2s+HUVECs, respectively.^32^ 96‐well round bottom ultra‐low attachment (ULA) plates were used for cell culturing. After 4 days of incubation of the cell suspension at 37°C with 5% CO_2_, through self‐aggregation, the cells naturally developed the liver microtissue compartments.^32^ DAPI (4′, 6‐diamidino‐ 2‐phenylindole) staining was administered to evaluate cell nuclei in microtissue samples according to the manufacturer's instructions. Additionally, haematoxylin and eosin staining were performed to analyse the morphological characteristics of the microtissue.

### 2.6 Fibrosis induction by free fatty acids (FFAs)

FFAs made of palmitic acid (PA) and oleic acid (OA) were administrated to the spheroids to evaluate the efficiency of liver fibrosis induction.^33^ OA (0.66 mm) and PA (0.33 mm) were combined in methanol and then frozen at 80°C before being used. 10% bovine serum albumin was added to the solution to facilitate their absorption into the hepatocytes. The solution was diluted (1:10) with a complete medium the next day. A cell culture medium containing FFAs was exchanged every other day for 4 days.

After fatty liver induction on day 4, the formed microtissues were subjected to treatment by three groups of substances; including free SIM, liposome and SIM‐LipoNPs. Treated groups were incubated for another 4 days and examined on the 8th day of cell culture.

### 2.7 Assessment of intracellular lipid accumulation

The lipid accumulation levels in the spheroids were assessed using oil red O (ORO). In brief, Spheroids were incubated with an ORO working mixture for 10–15 min after they had been washed by PBS and fixed in 4% paraformaldehyde solution. A light microscope was used for oil red O imaging, and a plate reader (FLUOstar Omega®, BMG Labtech) at 518 nm was applied to evaluate absorbance.

### 2.8 Dose finding design

To investigate the optimum dosage, we first examined 0.5 mg/mL, 5 mg/mL, 10 mg/mL and 50 mg/mL of Liposome, free SIM and SIM‐LipoNP, respectively. MTT assay was applied to determine the relative viability quantitatively. The determination of the appropriate dosage of SIM is informed by prior evidence showing decreased levels of fatty liver‐associated markers, including aspartate aminotransferase (AST), TNF‐α and IL‐6, following statin administration.^20^, ^22^

### 2.9 ROS evaluation

ROS expression levels of the samples were evaluated using the specific assay kit (Abcam). The spheroids were first rinsed with PBS and then exposed to dichlorodihydrofluorescein diacetate (DCFDA) in a 10% supplemented 1X buffer for 30 min at 37°C in the dark. Cytoplasmic ROS convert DCFDA to Dichlorofluorescein (DCF), a highly fluorescent compound. The fluorescence intensity of DCF was then checked using a microplate reader (excitation wavelength = 488 nm, emission wavelength = 535 nm) (FLUOstar Omega®, BMG Labtech, Germany).

### 2.10 Cell viability and proliferation

The viability of the spheroids' cells was measured with a LIVE/DEAD assay solution with fluorescent dyes applied. The solution was made of fluorescein diacetate (FDA) for staining live cells and propidium iodide (PI) for staining dead cells. Spheroids were exposed to the dye mixture (5 mg/mL and 2 mg/mL, respectively) for 5 min and subsequently washed with PBS. Fluorescent microscopy was employed to capture cellular images depicting green and red fluorescence signals.

The proliferation of cells was measured using the 3‐(4, 5 dimethyl‐2‐thiazolyl)‐2, 5‐diphenyl tetrazolium bromide (MTT) assay (M5655, Sigma‐Aldrich) after 8 days of cultivation. 200 μL of a 0.5 mg/mL MTT solution was added to the wells, incubating for 3.5 h at 37°C. 100 μL of dimethyl sulfoxide (DMSO; Merck) was used to replace the working solution in each well. Using a plate reader (FLUOstar Omega®, BMG Labtech), the stained spheroids' optical densities (ODs) were measured at 570 nm.

### 2.11 NO assay

The expression of NO was evaluated using the NO Assay Kit (Navand Salamat Co. Iran) following the manufacturer's instructions based on the method described by Su et al.^34^ Briefly, the quantification of nitrite (NO2‐) present in the culture medium is conducted by combining it with an equivalent amount of Griess reagent. Subsequently, the mixture is left to incubate at room temperature for duration of 15 mins. The amount of light absorbed by the sample at a wavelength of 540 nanometres was measured. The concentration of NO was then calculated by comparing the absorbance of the sample to a standard curve.

### 2.12 RNA extraction and quantitative real‐time polymerase chain reaction

The samples (*n* = 3 per group) were collected and kept in the deep freezer at −80°C for further analysis. The quantity of the mRNA levels of IL‐1 α, IL‐1 β, IL‐6 and TNF‐α as pro‐inflammatory cytokines, Krüppel‐like Factor 2 (KLF2) as the element playing a role in SIM‐KLF2‐NO signalling, and COL 1 as the core ECM component contributed in fibrosis condition were assessed by quantitative reverse transcription real‐time polymerase chain reaction (qRT‐PCR). The related primers for each target gene were used with reverse transcription and PCR amplification (Table 1). Total RNA was collected using an RNeasy Plus Mini Kit following the manufacturer's instructions (Giagen). Complementary DNA (cDNA) synthesis was performed using the RevertAid H Minus First Strand cDNA Synthesis Kit (Thermo Scientific, USA) following the manufacturer's instructions. The resulting cDNA aliquots were stored at −20°C until further analysis. Real‐time PCR (RT‐PCR) was conducted using the SYBR® Premix Ex TaqTM II kit (Takara, Japan) on an Applied Biosystems StepOnePlus™ System (ABI, USA). The ΔΔCt method was employed to calculate the expression fold change. Glyceraldehyde 3‐phosphate dehydrogenase (GAPDH) served as the reference gene for normalization, and the findings were reported as fold changes of the threshold cycle (Ct).

**TABLE 1:** Primers used in this study.

Gene	Sequence (5′ → 3′)	Size (Bp)	T_m_ (°G)	Product size (Bp)
IL‐1 α	F‐GAAGTCAAGATGGCCAAAGTTC R‐GAGACAGATGATCAATGGAGGAA	22 23	58.33 58.24	97
IL‐1 β	F‐GAGCTCGCCAGTGAAATGA R‐TTCATCTGTTTAGGGCCATCAG	19 22	58.34 58.63	83
IL‐6	F‐GAGATGTCTGAGGCTCATTCTG R‐CCTGCACTTACTTGTGGAGAA	22 21	58.79 58.38	103
TNF α	F‐AGCCTCTTCTCCTTCCTGAT R‐CCAGAGGGCTGATTAGAGAGA	20 21	57.95 58.57	121
COL‐I	F‐CCCTGGAAAGAATGGAGATGAT R‐GAAACCTCTGTGTCCCTTCAT	22 21	58.07 58.08	136
KLF‐2	F‐ACGCACACAGGTGAGAAG R‐GCGTGAGCTCGTCTGAG	18 17	57.72 57.93	79

### 2.13 Statistical analysis

To collect all the data, a minimum of three repeats were employed. For the statistical analyses, One‐way and two‐way analyses of variance (anova, Tukey HSD as a post‐hoc test) were performed. A *p*‐value of 0.05 or less was considered statistically significant. Standard deviation is illustrated as error bars.

## 3 RESULTS

### 3.1 Characterization of SIM‐LipoNPs

**FIGURE 1:**
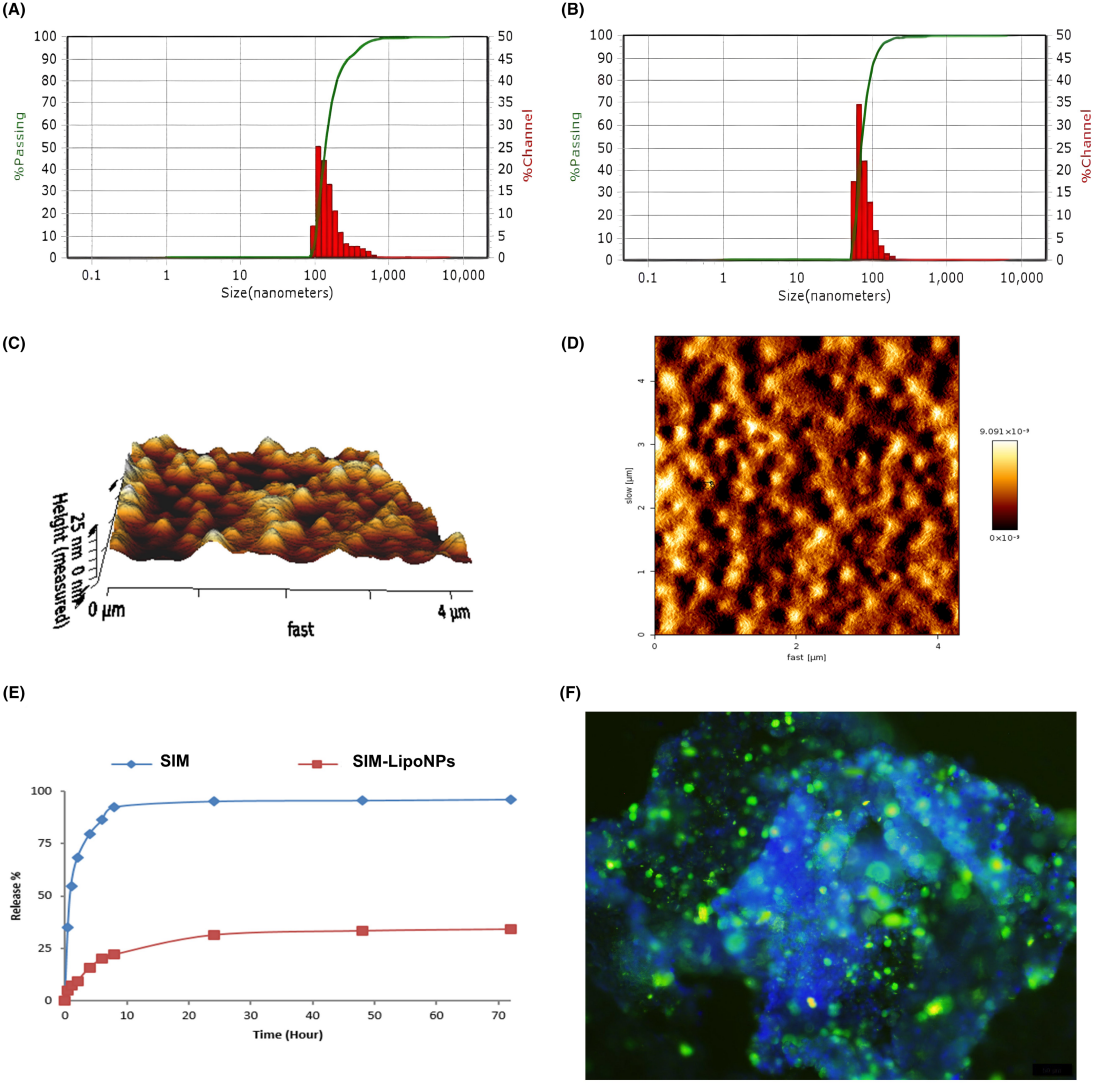
(A) The particle size distribution of empty liposome, (B) SIM‐LipoNP, (C) two‐dimensional AFM image of empty liposome, (D) 3D AFM image of empty liposome, (E) Release curve of free SIM and SIM‐LipoNP, (F) fluorescent imaging captures the delivery of FITC labelled LipoNPs to a DAPI‐stained complex multicellular liver microtissue. (scale bars: 50 μm).

The freeze‐drying technique is suitable for liposome formulation due to its ability to achieve elevated drug loading. The lyophilized product spontaneously assembles into a uniform liposome preparation upon adding an aqueous phase. Figure 1A,B showed the particle size analysis results of empty liposomes and the SIM‐LipoNPs, which were 71.6 nm and 139.5 nm, respectively. Loading SIM into liposome particles showed a slight increase in particle size. In addition, zeta potential results of the empty liposome and SIM‐LipoNPs were −52.9 ± 2.3 and 56.8 ± 1.1, respectively. The negative charge of the liposomes resulted from the lipid composition, which was not altered by the SIM loading. Two and three‐dimensional AFM images of empty liposomes are also presented in Figure 1C,D. Release curve of free SIM and SIM‐LipoNP is demonstrated in Figure 1E. Fluorescent imaging showed the delivery of FITC labelled liposomes to the multicellular liver microtissues, illustrated in Figure 1F.

The drug release profile was investigated using the dialysis bag method for 72 h in PBS (10 mM, pH = 7.4) at 350 rpm and a temperature of 37°C. As shown in Figure 1E, more than 90% of the free SIM was released from the dialysis bag after 8 h, while for the liposomal formulation, the release rate was significantly reduced, and only 35% of the drug was released after 72 h. The present release pattern is due to the interaction of drug molecules with the liposomal membrane and the drug concentration gradient on both sides of the membrane, resulting in slow release over time.

### 3.2 Fatty liver microtissue formation and viability

**FIGURE 2:**
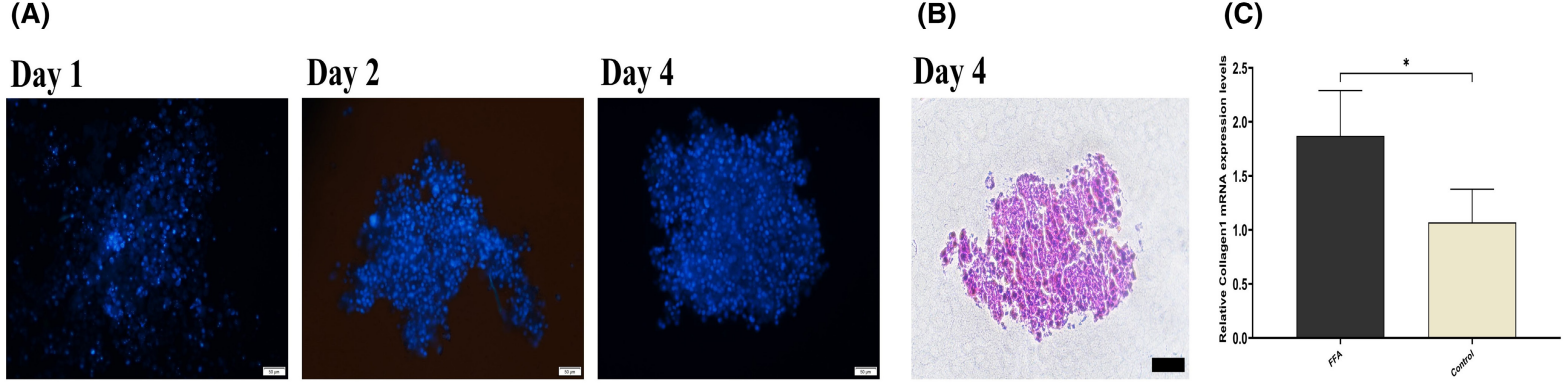
(A) DAPI staining exhibits nuclear morphology of micro tissue cells on days 1, 2 and 4. (scale bars: 50 μm) (B) Haematoxylin and eosin staining of liver microtissue on day 4. (scale bars: 100 μm) (C) Collagen I expression of microtissues on day 4 to validate the fibrotic changes. The data are presented as mean ± SD (*n* = 3 for each group). The symbol * signifies *p* value <0.05.

**FIGURE 3:**
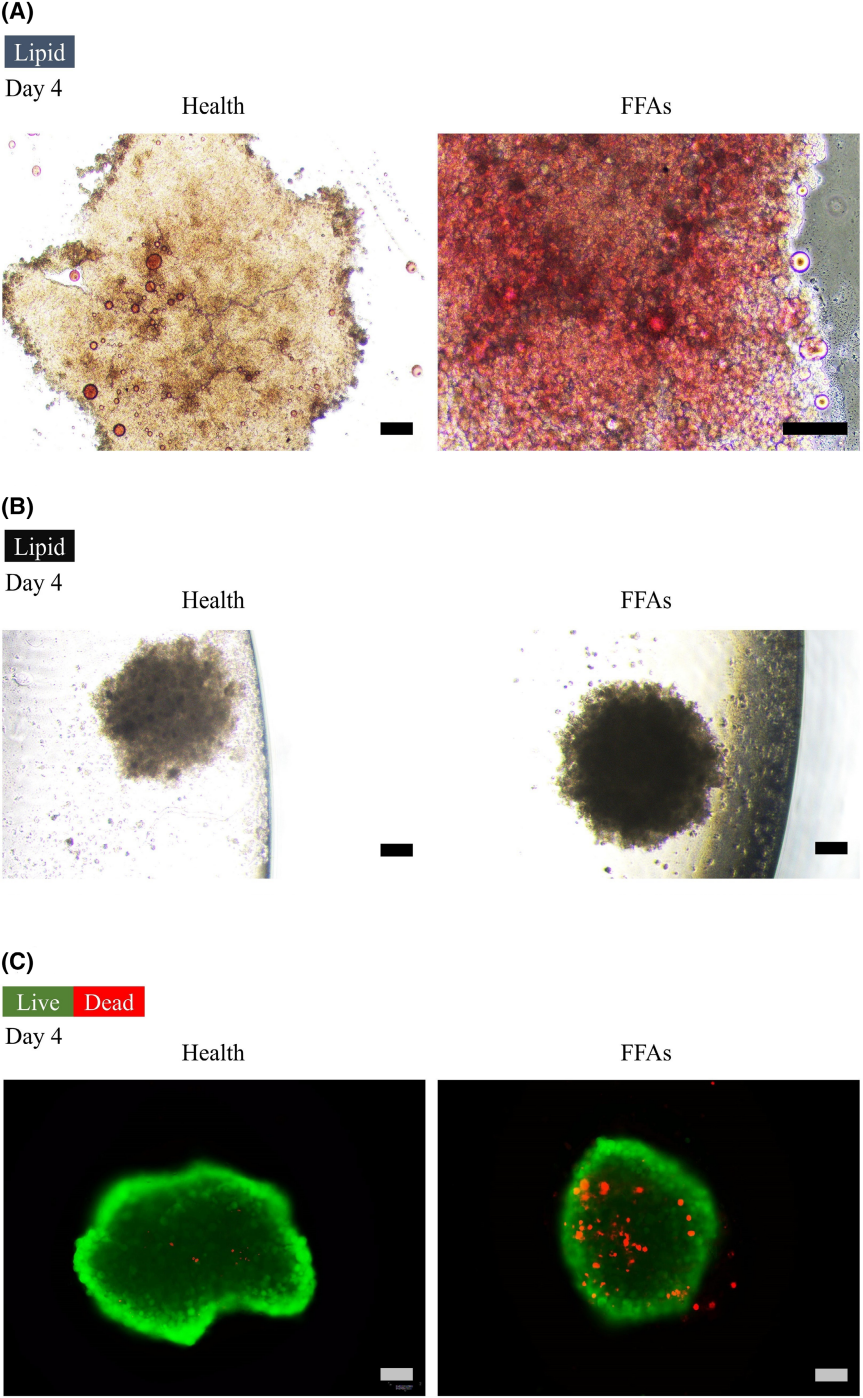
(A) Accumulated lipid droplets (Oil Red O staining). (scale bars: 50 μm), (B) Accumulated lipid droplets (light microscope) (scale bars: 100 μm), (C) Live (green)/dead (red) assay and quantification of dead cells on 4th Day. (scale bars: 100 μm).

A multicellular model consisting of HepG2, LX‐2 and HUVECs was developed to mimic the natural progression of fibrosis (Figure 2). Oil Red O staining was used to confirm lipid accumulation in liver microtissue on the 4th day of culturing (Figure 3A). Lipid droplet formation was investigated using a light microscope (Figure 3B), confirming the formation of lipid‐accumulated hepatocytes by adding FFAs. To qualitatively assess the viability of the fatty spheroids relative to the controls, the LIVE/DEAD assay solution confirmed the increased number of dead cells during fibrosis process after 4 days of FFA treatment (Figure 3C).

### 3.3 Dose finding

**FIGURE 4:**
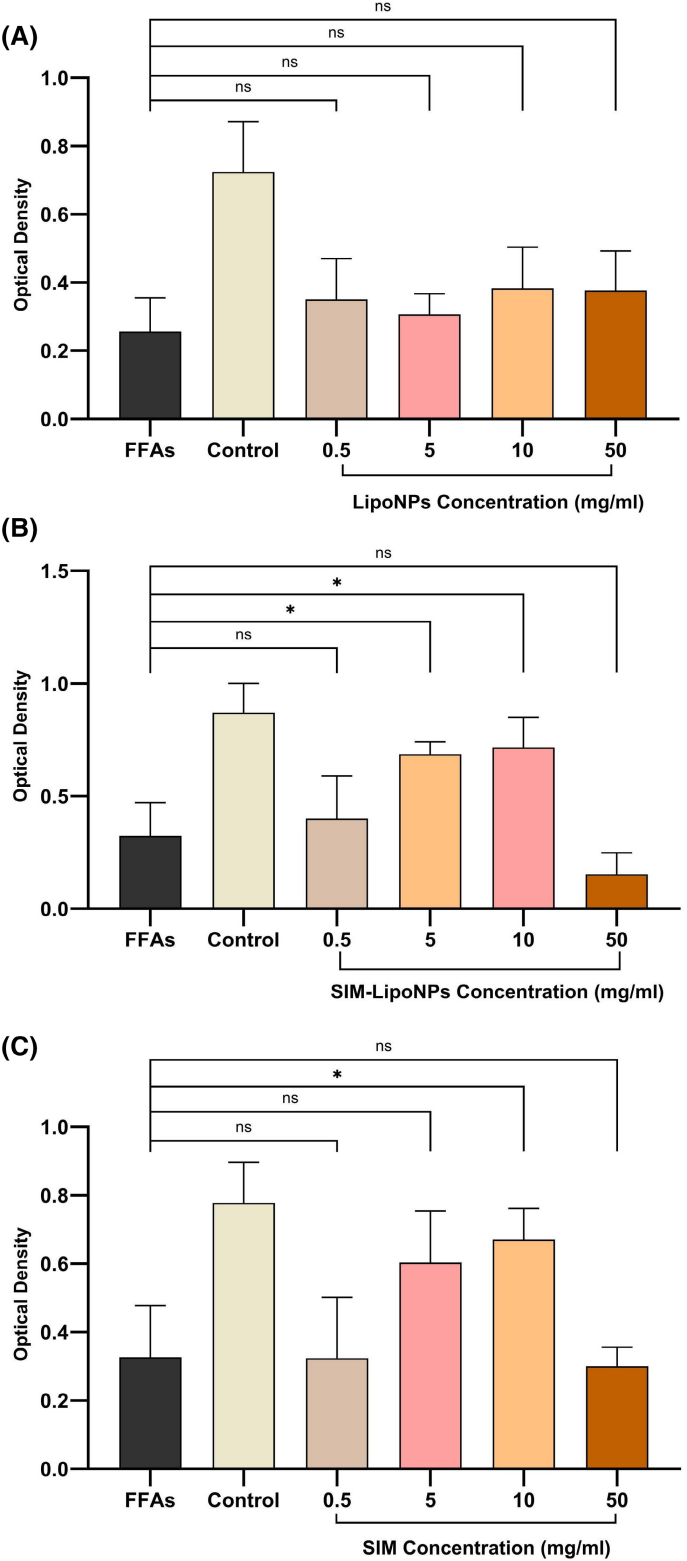
The cell‐proliferative capacity of treatment with (A) liposome, (B) simvastatin and (C) liposomal simvastatin was measured by MTT assay on 4th Day. The data are presented as mean ± SD (*n* = 3 for each group). The symbol * signifies *p* value <0.05, and the symbol **p value <0.01.

To further confirm the biocompatibility of the spheroids, the mitochondrial activity of the hepatocytes in all groups was assessed using the MTT assay (Figure 4). After 8 days of cell culturing, despite replacing media every other day, optimum density was lower in all subjected groups compared with control. It was shown that at the absorbance of 570 nm, optimum density was highest in the control group, and the SIM‐LipoNP at the dosage of 5 mg/mL and 10 mg/mL had the most similar results to the positive control and was significantly higher than the FFAs (*p* value <0.05).

**FIGURE 5:**
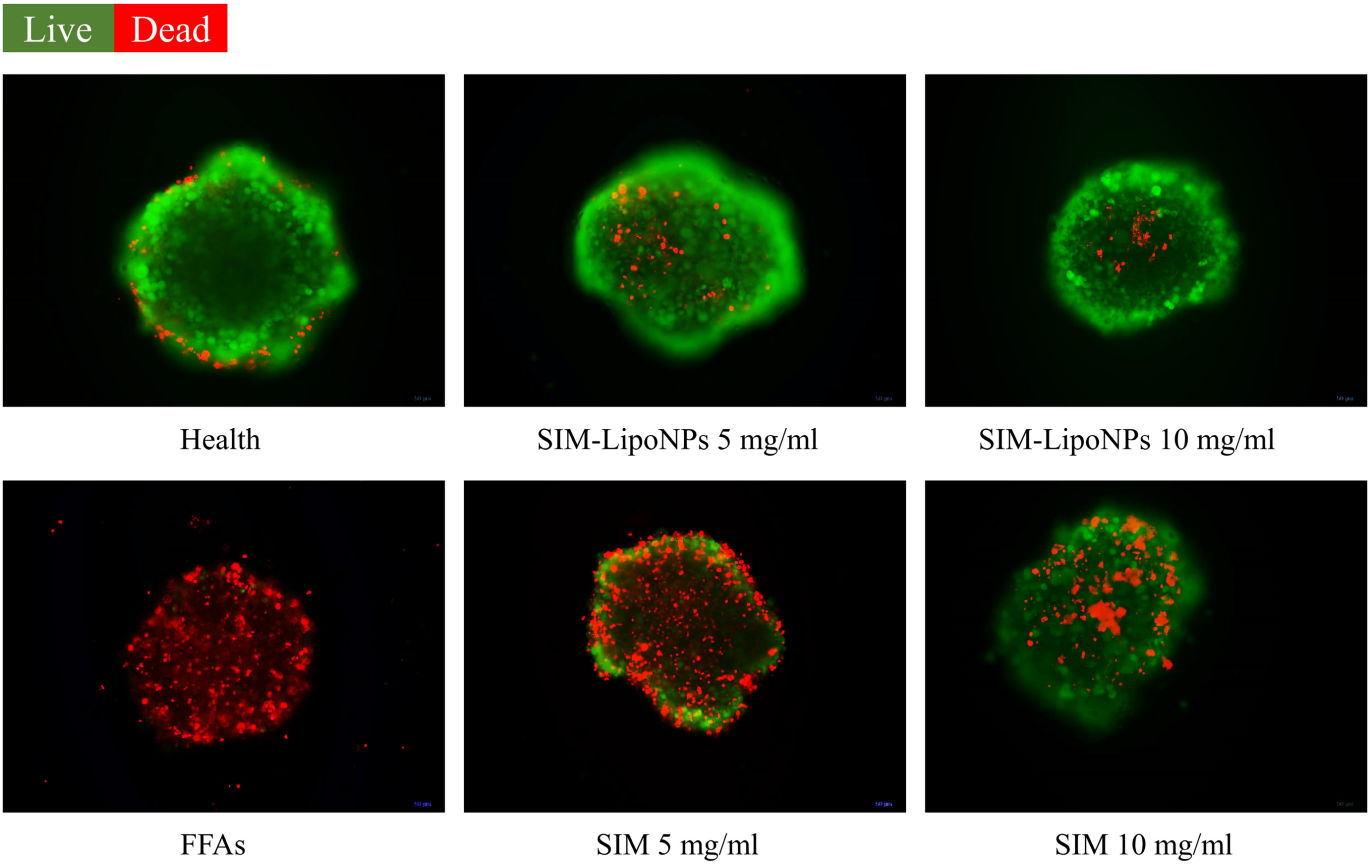
Cell viability within the microtissue was assessed. Green‐fluorescent cells indicated living cells, while red‐fluorescent cells indicated cell death on 8th Day. (scale bars: 100 μm).

The number of fluorescent microscopes presenting dead/live cells (red/green points) in the negative controls was significantly higher than in the positive controls (Figure 5). While 5 mg/mL SIM couldn't present a significant impact on preventing cell death during fibrosis process, SIM‐LipoNP showed promising results of high viability at this dosage. The quantitative analysis of dead cells revealed an average of 84.69 ± 12.23 in the FFA group, 8.91 ± 2.68 in the control group, 51.69 ± 7.33 in the SIM (5 mg/mL) group, 24.76 ± 3.86 in the SIM‐LipoNP (5 mg/mL) group, 38.61 ± 15.07 in the SIM (10 mg/mL) group and 13.37 ± 8.92 in the SIM‐LipoNP (10 mg/mL) group. This outcome was in line with the MTT assay's findings.

### 3.4 SIM‐LipoNP activated KLF2‐NO pathway

**FIGURE 6:**
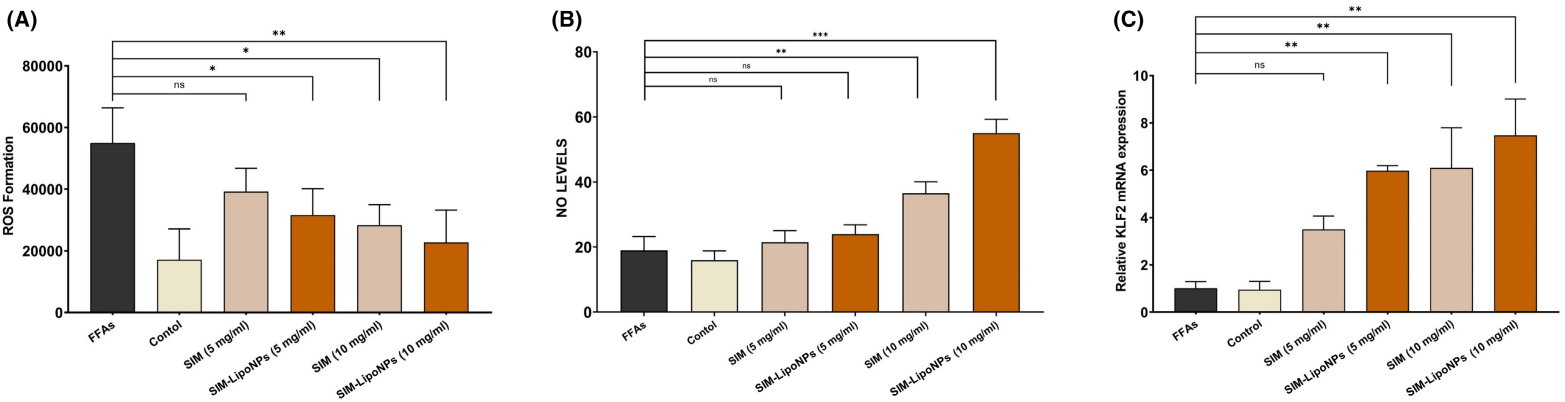
(A) ROS activity, (B) No levels and (C) KLF2 gene expression in the microtissue. The data are presented as mean ± SD (*n* = 3 for each group). The symbol * signifies *p* value <0.05, and the symbol **p value <0.01.

By incorporating both endothelial cells and HSCs in microtissue samples, we facilitated a more comprehensive exploration of the impact of SIM and SIM‐LipoNP on the expression of KLF2. Both SIM and SIM‐LipoNP at 10 mg/mL, and SIM‐LipoNP at 5 mg/mL, significantly increased KLF2 expression (*p* < 0.05, *p* < 0.005 and *p* < 0.05, respectively) (Figure 6). These data were confirmed by NO expression data, which showed significant increases in response to 5 and 10 mg/mL SIM‐LipoNP (*p* < 0.05 and *p* < 0.005, respectively) and 10 mg/mL SIM (*p* < 0.05). Notably, at 10 mg/mL, SIM‐LipoNP had a more substantial impact on NO and KLF2 expression compared to SIM.

### 3.5 SIM‐LipoNP protects against fibrosis‐induced oxidative damage

To understand the possible antioxidative effect of the SIM‐LipoNP compared to SIM, we examined the ROS levels in the Fibrosis models responding to either 5 or 10 mg/mL dosage. ROS expression levels showed a significant decrease by about 50% in response to 10 mg/mL SIM‐LipoNP (*p* value<0.005) and a slight but significant reduction in 10 mg/mL free SIM and 5 mg/mL SIM‐LipoNP (*p* values <0.05) (Figure 6).

### 3.6 Reduced pro‐inflammatory factors in the SIM‐LipoNP treated models

**FIGURE 7:**
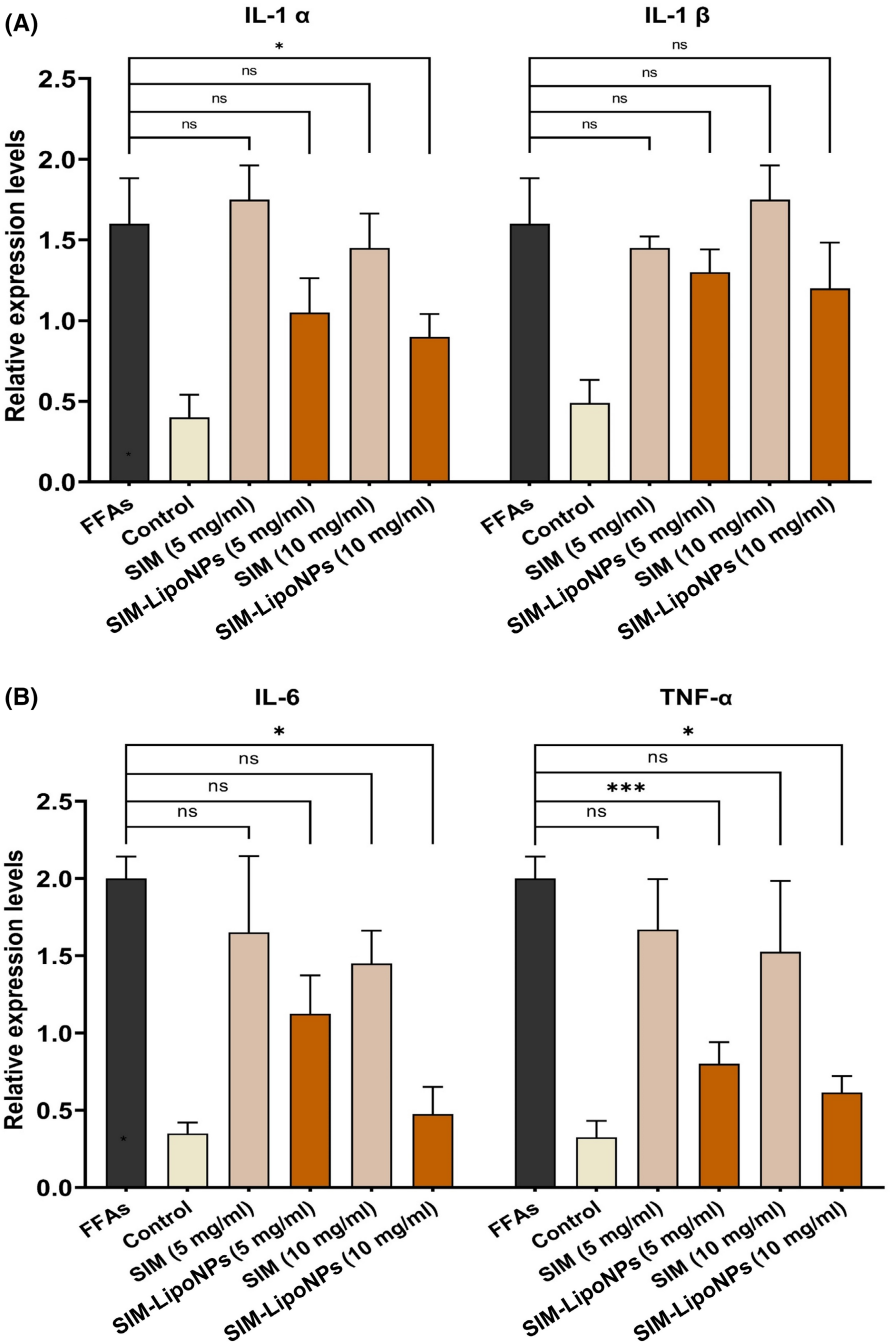
Gene expression of pro‐inflammatory cytokines in human liver microtissue including (A) IL‐1 α, IL‐1 β and (B) IL‐6, and TNF‐α. The data are presented as mean ± SD (*n* = 3 for each group). The symbol * signifies *p* value <0.05, and the symbol **p value <0.01.

NAFLD condition causes a significant increase in pro‐inflammatory factors, including IL‐1 α, IL‐1 β, IL‐6 and TNF‐ α. The expression of the pro‐inflammatory factors was compared in both the control and treated groups to investigate the protective effects of both SIM and SIM‐LipoNP in the fatty liver models. A look at these early (IL‐1 α and IL‐1 β) and late (IL‐6 and TNF‐α) phase pro‐inflammatory cytokines revealed significantly reduced secretion of IL‐1 α, IL‐6 and TNF‐α by about 10%, 60% and 50%, respectively, in 10 mg/mL SIM‐LipoNP exposed spheroid models (*p* values <0.05) (Figure 7). For IL‐1 and IL‐6 expression, only the SIM‐LipoNP (10 mg/mL) group showed significant reduction. Both the 10 mg/mL and 5 mg/mL SIM‐LipoNP groups significantly reduced TNF expression. These findings indicate that the SIM‐LipoNP (10 mg/mL) group was the most effective among those tested.

### 3.7 Collagen I expression profile upon SIM‐LipoNP administration

**FIGURE 8:**
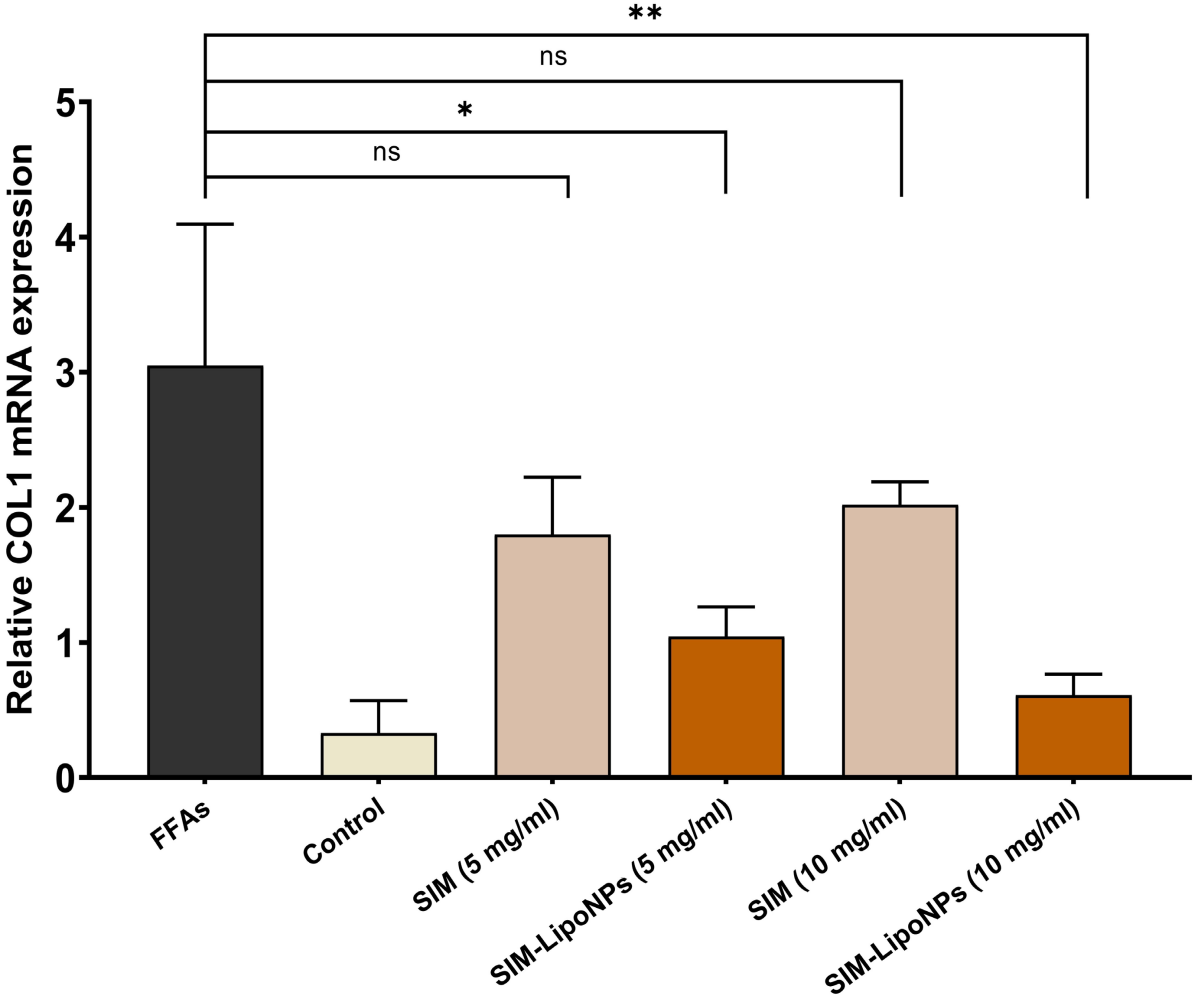
Collagen I expression of microtissues. The data are presented as mean ± SD (*n* == 3 for each group). The symbol * signifies *p* value <0.05, and the symbol **p value <0.01.

An effective substance against fibrosis presses might reduce the overgeneration of collagen I, the main ECM component that is over‐generated in liver fibrosis. The findings of this study demonstrated that only the SIM‐LipoNP group exhibited a significant effect on Collagen I (Col 1) mRNA expression, while the SIM group failed to induce significant changes. Although SIM‐LipoNP at 5 mg/mL was effective, the 10 mg/mL concentration had a more pronounced effect. Our results revealed that untreated fibrotic control samples had Collagen I mRNA levels five times greater than that of the group receiving 10 mg/mL SIM‐LipoNP and three times greater in 5 mg/mL SIM‐LipoNP (*p* values <0.05) (Figure 8).

## 4 DISCUSSION

Despite its global prevalence and lack of definitive treatment, NAFLD research suffers from limited study models. While animal models have driven understanding, inherent genetic and epigenetic disparities between species hinder their efficacy in drug discovery. This contributes to the high failure rates (exceeding 80%) observed in clinical trials, primarily due to efficacy (60%) and toxicity (30%) concerns.^35^, ^36^ Rigorous validation and translational promise of novel preclinical tools have prompted the US food and drug administration to waive mandatory animal testing for certain human trials. This landmark decision marks a paradigm shift in drug discovery, streamlining development and facilitating advancements in therapeutic research.^37^ Developing in vitro models that accurately emulate human physiology is critical for comprehending disease processes, facilitating mechanistic research and expediting the evaluation of novel therapeutic approaches. Herein, it is revealed how a multilinear microtissue representing fibrosis liver could be an optimum choice to investigate the effect of treatment strategies and drug screening primarily. The remarkable feature of this model is the co‐culturing of primary human hepatocytes with non‐parenchymal cells, including endothelial cells and HSCs in the same proportion (parenchymal 60% vs. non‐parenchymal 40%) as found in human liver tissue.^32^ In agreement with earlier reports, HepG2 co‐culturing with LX‐2 enhanced the density of the models, while the HepG2s formed in spheroids showed not to be able to synthesize ECM, only sporadically adhering to one another.^38^ Moreover, co‐culturing HepG2 cells with HUVECs resulted in a more distinct stimulation of liver pathology.^39^ Involving a proper ratio of HepG2,  LX‐2 and HUVECs in this study provided a circular shape spheroids without a necrotic core, highlighting the fact that circularity is a crucial feature to maintaining the linear gradient of oxygen tension that aids tissue haemostasis.^40^, ^41^

This investigation with SIM‐LipoNP formulation aimed to find an alternative for free SIM against NAFLD to overcome its limitations. In addition to reducing hepatic side effects at lower dosages, for a durable intravenous injection that promotes blood circulation to the region of the fatty liver, SIM in the form of nanoliposomes can to be an improved alternative. To provide sufficient drug delivery to the injury site, the nanoliposomes may also retain the bulk of the substance payload within its vesicles during circulation.^22^ Additionally, this formulation showed a comparatively slight burst release, suggesting that a significant amount of the medication may be effectively administered at the site of damage.

As shown in Figure 4, while free LipoNPs improved cell viability compared to FFAs (the positive control group), the difference was not statistically significant. Additionally, free simvastatin increased cell viability with rising concentrations up to 10 mg/mL. Similarly, the SIM‐LipoNP group demonstrated increased cell viability with higher SIM‐LipoNP concentrations, also up to 10 mg/mL. Although the viability percentages were higher in the SIM‐LipoNP group compared to the SIM group, this difference was not statistically significant. This may be attributed to the encapsulation of simvastatin in lipid nanoparticles, which potentially altered its pharmacokinetics, biodistribution and cellular interactions, resulting in different effect profiles.

Furthermore, the observed decrease in cell viability at 50 mg/mL in both SIM and SIM‐LipoNP groups, is likely due to the cytotoxicity of simvastatin at higher concentrations. A recent study showed that simvastatin significantly induces cell death in a dose‐dependent and time‐dependent manner in MCF‐7 and MDA‐MB‐231 cell lines.^42^ Thus, in our study, the 50 mg/mL concentration of simvastatin may exceed its half‐maximal inhibitory concentration (IC50) in the microtissue model, leading to reduced cell viability. The underlying pathological pathways were further experimented with to investigate the protective effects of SIM and SIM‐LipoNP.

In this research, we investigated the effects of SIM‐LipoNP administration on the behaviour of the KLF2‐NO signalling pathway by analysing the expression levels of KLF2 and NO. Consistent with this data, 5 mg/mL SIM‐LipoNP administration indicated a significant elevation in KLF2 expression levels, while the same result wasn't observed when using the same dosage of free SIM. Based on earlier reports, SIM specifically activates the KLF2‐NO pathway in endothelial cells, aligning with the significant NO increase after 10 mg/mL of either SIM‐LipoNP or SIM administration in this study. Also, by administering SIM, the induced expression of KLF2 resulted in the release of vascular endothelial growth factor (VEGF) from HSCs and enhancement of the liver sinusoidal endothelial cells (LSECs) characteristics.^43^ In an in‐vivo study, the SIM‐KLF2‐NO pathway was reported to decrease hepatic vascular tone and ameliorate the dysfunction of liver sinusoidal endothelial cells in rats with cirrhosis.^16^

The results of our study showed that at its lower doses (5 mg/mL), SIM‐LipoNP can induce marked antioxidative regulation in the Fibrosis models. Previous studies documented the substantial antioxidative effects of SIM in a murine model of experimental NAFLD.^17^, ^18^ The antioxidative effect of SIM in this fibrosis liver microtissue model is probably induced by KLF2‐NO signalling in LSECs which upregulates the expression of Chemokine (C‐X‐C motif) ligand 16 (CXCL16) on LSECs. This pathway has been shown to lead to shifting HSCs from activation to quiescence.^19^, ^20^, ^44^, ^45^ This data is further confirmed by the results from Col‐1 expression levels, as the main component over‐generated upon HSCs activation showed a significant decrease in liposomal SIM administrated groups. Moreover, reports from Wang et al. suggested that Liposome‐encapsulated statins show a considerable reduction of type I/III collagen content in the wound scar.^46^

In the same way, based on previous reports, NO exhibits significant protective potential against inflammatory and oxidative stress responses: attenuating pro‐inflammatory cytokines like TNF‐α and IL‐1 prevents endothelial damage. Additionally, sustained NO production demonstrably decreases ROS and further curbs pro‐inflammatory cytokine generation, mitigating overall liver injury. Furthermore, inadequate NO levels contribute to the accumulation of ROS, which subsequently induces the expression of TNF‐α and exacerbates liver inflammation.^47^, ^48^

As previously mentioned, pro‐inflammatory cytokines, such as IL‐1α, IL‐1β, TNFα and IL‐6, are involved in liver inflammation, steatosis, fibrosis and cancer development.^49^ Overgeneration of TNF‐α and IL‐6 can lead to HSC activation, which results in an extracellular matrix containing high levels of Col‐1 accumulation that deteriorates fatty liver condition.^50^ Pro‐inflammatory cytokines secreted by the microtissues upon treatment of either SIM‐LipoNP or SIM alone were examined. While simvastatin offered limited anti‐inflammatory effects in fibrosis models, a noticeable regulation in the inflammatory pathways was observed with SIM‐LipoNP administration. These data align with the results from the study by Rakshit et al. announcing that 10 mg/mL liposomal SIM administration upon atherosclerosis 3D foam cells model illustrated significant modulation in pro‐inflammatory factors expression.^22^

## 5 CONCLUSION

Here, it is sought to investigate the use of liposomes for SIM delivery to provide a higher efficacy. Together, this study revealed that SIM can be efficiently encapsulated in liposomes, and the Liposomal form has improved fatty liver condition in vitro. The findings show that Liposomes containing SIM are more effective in treating Fibrosis models than the free drug. Confirmed by the results of this investigation, SIM‐LipoNP at its lower dosages could be competitive with a higher dosage of free SIM, probably by activation of the KLF2‐NO signalling pathway. This study presents evidence supporting the potential effectiveness of liposomes in overcoming challenges associated with SIM delivery. However, there is limited data on the efficacy of SIM within targeted liposomal delivery systems specifically tailored for precise liver delivery. Finally, the effectiveness of liposomal statins is also poorly understood due to a lack of evidence from in‐vivo studies. Therefore, more investigation is required to assess the pharmacokinetic and pharmacodynamics properties of SIM‐LipoNP in humans.

## AUTHOR CONTRIBUTIONS

**Shima Parsa:** Conceptualization (equal); formal analysis (equal); funding acquisition (equal); investigation (equal); methodology (equal); validation (equal); writing – original draft (equal). **Maryam Dousti:** Investigation (equal); methodology (equal). **Nasim Mohammadi:** Investigation (equal); software (equal). **Mozhgan Abedanzadeh:** Investigation (equal). **Niloofar Dehdari Ebrahimi:** Investigation (equal); visualization (equal); writing – original draft (equal). **Mahintaj Dara:** investigation (equal); Formal analysis (equal). **Mahsa Sani:** Data curation (equal); investigation (equal); software (equal). **Muhammad Nekouee:** Investigation (equal). **Samira Sadat Abolmaali:** Investigation (equal). **Farnaz Sani:** Conceptualization (equal); investigation (equal); methodology (equal); supervision (equal). **Negar Azarpira:** Conceptualization (equal); funding acquisition (equal); project administration (equal); supervision (equal).

## FUNDING INFORMATION

This research received support solely from the authors. We affirm that no additional external funding was utilized, and there were no competing interests or conflicts related to funding.

## CONFLICT OF INTEREST STATEMENT

The authors declare that they have no conflict of interests.

## Data Availability

The datasets used and analysed during the current study are available from the corresponding authors on reasonable request.
