# Correction to “The Effects of Simvastatin‐Loaded Nanoliposomes on Human Multilineage Liver Fibrosis Microtissue”

**DOI:** 10.1111/jcmm.71360

**Published:** 2026-09-30

S. Parsa , M. Dousti , N. Mohammadi , M. Abedanzadeh , N. Dehdari Ebrahimi , M. Dara , M. Sani , M. Nekouee , S. S. Abolmaali , F. Sani , and N. Azarpira , “The Effects of Simvastatin‐Loaded Nanoliposomes on Human Multilineage Liver Fibrosis Microtissue,” Journal of Cellular and Molecular Medicine 28, no. 13 (2024): e18529. 10.1111/jcmm.18529.PMC1123464738984945

In Figure 3C, the red fluorescence channel image in the FFA group was inadvertently merged incorrectly during figure assembly. The correct red channel image has now been merged with the corresponding green channel image and the corrected panel has been replaced in Figure 3C. This correction does not affect the conclusions drawn in the study.

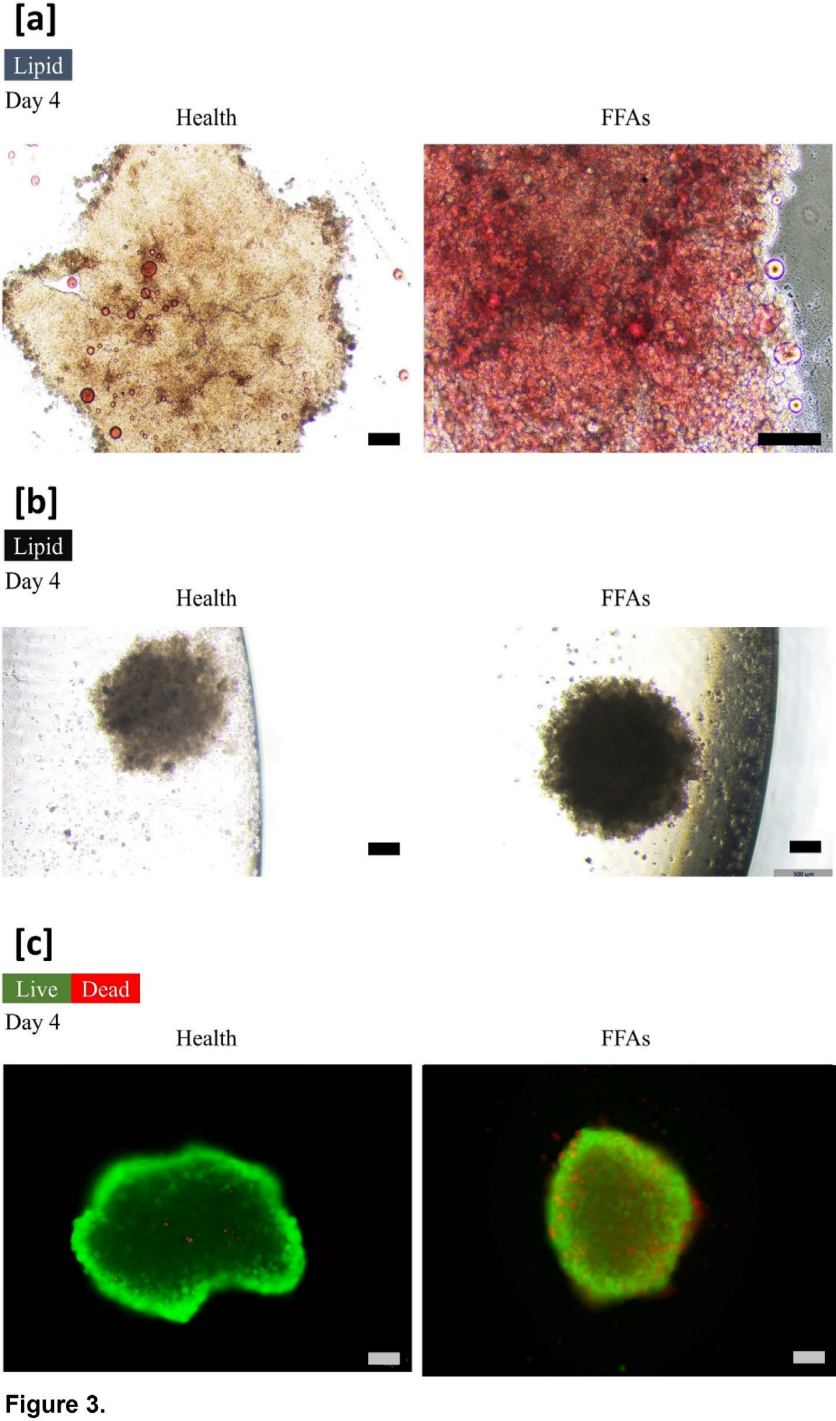

We apologize for this error.

